# A Serum Vitamin D Level <25nmol/L Pose High Tuberculosis Risk: A Meta-Analysis

**DOI:** 10.1371/journal.pone.0126014

**Published:** 2015-05-04

**Authors:** Junli Zeng, Guannan Wu, Wen Yang, Xiaoling Gu, Wenjun Liang, Yanwen Yao, Yong Song

**Affiliations:** 1 Department of Respiratory Medicine, Jinling Hospital, Southern Medical University (Guangzhou), Nanjing, China 210002; 2 Department of Respiratory Medicine, Jinling Hospital, Nanjing University School of Medicine, Nanjing, China 210002; Fundació Institut d’Investigació en Ciències de la Salut Germans Trias i Pujol. Universitat Autònoma de Barcelona. CIBERES, SPAIN

## Abstract

**Background:**

Low serum Vitamin D is considered to be associated with tuberculosis while the “dangerous” level was not clear. The aim of this study was to identify the association between tuberculosis and serum Vitamin D levels via synthesis of available evidence.

**Methods:**

A search of EMBASE, Medline, ISI Web of knowledge, and Pubmed was conducted. The number of subjects of tuberculosis and no-tuberculosis groups in four Vitamin D range. Meta-analyses were performed and presented by odds ratios (ORs) and corresponding 95% confidence intervals (CIs).

**Results:**

A total of 15 studies involving 1440 cases and 2558 controls were included. A significantly increased risk of tuberculosis was found in two ranges: ≤ 12.5 nmol/L: pooled OR = 4.556, 95% CI = 2.200-9.435; 13-25 nmol/L: pooled OR = 3.797, 95% CI = 1.935-7.405. No statistically significant risk of tuberculosis was found in the range of 26–50 nmol/L (pooled OR = 1.561, 95% CI =0.997-2.442). In range 51–75 nmol/L, no positive association was found (pooled OR =1.160, 95% CI = 0.708-1.900).

**Conclusions:**

This study found that a serum Vitamin D level ≤ 25 nmol/L was significantly associated with an increased risk of tuberculosis while the range of 51–75 nmol/L was not. The range 26-50nmol/L posed potential high tuberculosis risk. Future large-scale, well-designed studies are needed to verify these results.

## Introduction

Tuberculosis (TB) remains a major challenge to global public health. [1] In addition to HIV infection, other factors that contribute to susceptibility and progression of TB have not been well defined. [1] It has been suggested that susceptibility to TB is associated with host immune response and could be influenced by environmental and genetic factors or by gene-environment interactions. [2]

Vitamin D, an immunomodulatory effector, has been proven to play a critical role in inducing antimycobacterial activity which is accomplished by inhibiting the growth of Mycobacterium tuberculosis (MTB) and up-regulating protective innate host responses.[3, 4] It has been shown that Vitamin D modulates monocyte-macrophage activity by binding to Vitamin D receptors (VDRs), which are responsible for both intracellular replication of MTB and the destruction of MTB by acting as antigen presenting cells (APCs).[5] Previous clinical studies have indicated that “Vitamin D deficiency” is associated with an increased risk of tuberculosis; however, the criteria for “Vitamin D deficiency” differed among these studies. Some of these studies used a concentration of 25 hydroxycholecalciferol (25-(OH)D3) < 50 nmol/L as a cutoff value for Vitamin D deficiency [6–8] while some other studies used a concentration of 25-(OH)D3 below 25 nmol/L.[9, 10] Some studies also used other criteria, including 30 nmol/L and 62.5 nmol/L in some studies. Meanwhile, although a meta-analysis in 2008 [11] found that low serum Vitamin D levels are associated with a higher risk of active tuberculosis, the exact range of serum Vitamin D for low serum Vitamin D was not defined. Explicit “dangerous” serum Vitamin D should be more practical in clinical and referable for future studies. Furthermore, a number of studies were published during the past six years in this field. Thus, we performed this meta-analysis to further define the precise range of serum Vitamin D that contributes to an increased TB risk. To the best of our knowledge, this is the first meta-analysis to verify serum Vitamin D range posing an increased TB risk.

## Methods

### Literature Search Strategy

A literature search of EMBASE, Medline, ISI Web of knowledge, and Pubmed up to February, 2015 was conducted. Search terms included tuberculosis, TB, serum Vitamin D, Vitamin D, Vitamin D deficiency and cholecalciferol. After obtaining all searching results, we eliminated unrelated publications by title reading and performed manual searches of citations from these original studies. To avoid replicated data in different publications from the same author or the same researching team, we further checked these remaining articles and none of the included studies were found to contain this problem.

### Study selection and Quality evaluation

Studies were included if they meet the following criteria: a) studies must be reported in English and have available full text; b) the organism studied must be Mycobacterium tuberculosis (MTB); c) studies must involve a TB group and a control group, investigate the association between serum Vitamin D concentrations and TB in humans and supply the number of participants in our defined concentration range or quite closing range. Exclusion criteria were: a) reviews or meta-analyses; b) fundamental studies of cells or animal models; c) the studies enrolled patients known to be immunosuppressed (eg, HIV-1 infection, pregnancy, or corticosteroid therapy). No special limitation of age, population or study design was defined in this study.

The Newcastle–Ottawa Scale [12], a validated technique for assessing the quality of observational and nonrandomized studies, was used to evaluate the quality of these studies. A star system was applied to evaluate studies based on three criteria: participant selection, comparability of study groups, and assessment of outcome or exposure. (S1 Appendix.)

### Data Extraction

Two reviewers (Zeng and Wu) independently collected data from each study. Disagreements were resolved by discussion between them and a third author was consulted to obtain a final decision if a consensus could not be reached between them. The following items were extracted from each study: First author, age of both groups, male percentage of both groups, nationality, ethnicity, source of controls, TB diagnosis standards and methods, number of TB patients and controls in different ranges of serum Vitamin D, study design, percentage of participants in each range, body mass index (BMI) in both groups and the treatment status of participants. Some studies offered detailed figures with obtainable data and we retrieved all available information from these figures. All included studies defined serum Vitamin D by 25-(OH)D3, which has been widely accepted as a better indicator of vitamin D status than 1,25(OH)2D3. [13] Thus, we used 25-(OH)D3 in this study.

### Statistical analysis

All statistical analyses were performed using STATA11.0 software (StataCorp, College Station, TX, USA). The strength of association was evaluated by odds ratios (ORs) with corresponding 95% confidence intervals (CIs). All meta-analyses were conducted by using a random-effects model. Heterogeneity among studies was assessed by the χ^2^ test and I^2^ statistic. [14]Subgroup analyses and meta-regression analyses were conducted. Sensitivity analyses were also conducted to determine whether an individual study affected the summary meta-analysis estimate. Publication bias was examined by Begg’s test and Egger’s test, and a Begg’s funnel plot was acquired. A value of “Pr > |z|” less than 0.05 or a value of “P > |t|” less than 0.05 was considered to be potential publication bias. The PRISMA 2009 Checklist was used for further validation of the meta-analysis.(S1 PRISMA Checklist.)

## Results

### Characteristics and quality of the Studies

**Fig 1:**
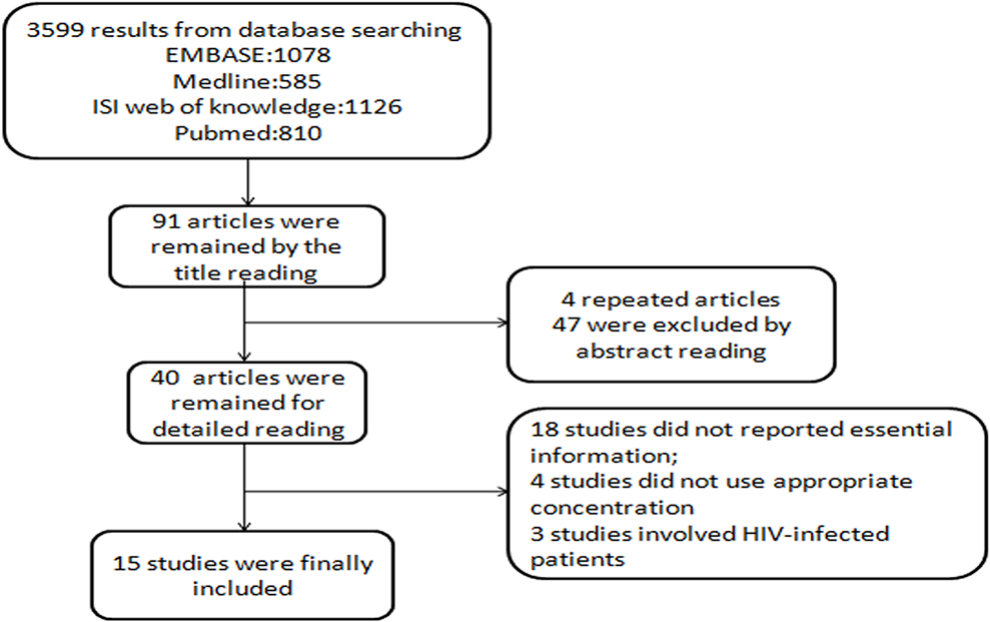
Flow diagram of study selection.

The study selection process is schematically presented in Fig 1. Based on the searching strategy, 3599 results were identified. 40 studies remained for detail reading. 20 potential studies were retrieved for more detailed evaluation. Three [15–17] of them just provided the data of patients complicated with HIV infection, and the appropriate data could not be extracted from the other two studies [18,19]. Finally, 15 articles published from 1985 through 2014 were included for further analysis.[6–10, 20–29] These 15 eligible studies included 1440 cases and 2558 controls. Eight [6–10, 20–22] of them only involved Asian participants and two [23, 24] only involved African subjects. Nine [6, 8–10, 20, 22, 23, 25, 26] studies involved adults only, and two [21, 27] studies involved children only. In addition, serum 25(OH)D3 was measured by different methods, including radioimmunoassay [7, 8, 10, 20, 22, 25, 26 28], chemiluminescence immunoassay [9, 29], liquid chromatography assay [6, 23], and Enzyme-Linked Immunosorbent Assay (ELISA) [21]. More detailed characteristics are presented in Table 1.

**Table 1:** Characters of included studies.

Reference	Population	Design	NO. of participants		Diagnosis criteria of TB	Source of Controls	Adults or Children
			TB	Control			
Grange et al 1985^**[**^ ^**20**^ ^**]**^	Indigenous Indonesian	Case-control	40	38	Smear-positive	health age matched subjects	Adults
Hong et al 2014^**[**^ ^**10**^ ^**]**^	Korean	Case-control	94	282	Positive biopsy culture; clinical symptoms; abnormal X-ray	random population sample	Adults
Ho-Pham et al 2010^**[**^ ^**9**^ ^**]**^	Vietnam	Case-control	166	219	Positive biopsy culture; typical symptom; typical radiograph	random population sample	Adults
Wilkinson et al 2000 ^**[**^ ^**7**^ ^**]**^	Gujarati	Case-control	103	42	Positive biopsy culture	household contacts of TB	*NA
Kim et al 2013^**[**^ ^**6**^ ^**]**^	Korean	Case-control	165	197	Positive biopsy culture; typical radiograph; respond to anti-TB therapy	health subject	Adults
Joshi et al 2013 ^**[**^ ^**21**^ ^**]**^	Indian	Case-control	25	50	Positive ^†^AFB smear microscopy	household contacts and health control	Adults
Koo et al 2012^**[**^ ^**22**^ ^**]**^	Korean	Case-control	116	82	Positive PCR test; granulomas and exudative effusion with ^‡^ADA>40 IU/L	volunteers with no history and present TB	Adults
Jubulis et al 2014^**[**^ ^**8**^ ^**]**^	Indian	Case-control	25	118	Positive biopsy culture; typical chest radiograph	no TB patients	children
Wejse et al 2007^**[**^ ^**23**^ ^**]**^	Guinea-Bissau	Case-control	362	949	WHO TB guidelines	random population sample	Adults
Nielsen et al 2010 ^**[**^ ^**25**^ ^**]**^	Greenland	Case-control	72	72	Positive biopsy culture; X-ray confirmed; IFN release test	random population sample	Adults
Gibney et al 2008^**[**^ ^**24**^ ^**]**^	Australian	Retrospectiveclinicalaudit	40	34	Microbiological evidence; response well to anti-TB therapy	a negative ^§^PPD test	*NA
Davies et al 1985 ^**[**^ ^**26**^ ^**]**^	White(84%) Indian(8%) Other (8%)	Case-control	40	40	Positive biopsy culture; pulmonary TB	family members of patients or health volunteer	Adults
Sita-Lumsden et al 2007 ^**[**^ ^**28**^ ^**]**^	^#^Mixed	Case-control	178	130	Positive biopsy culture	healthy contact	Both
Gray et al 2012^**[**^ ^**27**^ ^**]**^	Australian	Retrospectiveclinicalaudit	11	317	^§^PPD positive; typical radiograpy	no TB patients	children
Arnedo-Pena et al 2015^**[**^ ^**29**^ ^**]**^	#Mixed	Prospective cohort study	3	520	^§^PPD positive and ※QFT-GIT positive	Contact of pulmonary TB patients	Both

Note:

Abbreviations:

*NA: not available

^†^AFB:acidfast bacilli

^‡^ADA: adenosinedeaminase level

^§^PPD: purified protein derivative(of tuberculosis)

^#^Mixed: patients in this article were classified by skin color while the ethnic information was not provided

^※^QFT-GIT: QuantiFERON-TB Gold In-Tube test

Because these included studies were observational and nonrandomized, the quality of the primary studies were evaluated by the Newcastle–Ottawa Scale. [12] Five studies received a score of 5, three studies received a score of 6, and seven studies received a score of 7 or 8. Further details regarding the scoring are presented in Table 2.

**Table 2:** Quality assessment of included studies using Newcastle-Ottawa Scale.

Study	Selection				Comparability		Exposure			Score
J. M. Grange^**[**^ ^**20**^ ^]^	1			1	1		1	1		5
J. Y. Hong^**[**^ ^**10**^ ^**]**^	1	1	1	1		1	1	1		7
Lan T Ho-Pham^**[**^ ^**9**^ ^**]**^	1	1	1	1	1	1	1	1		8
Nina O. Nielsen^**[**^ ^**25**^ ^**]**^	1	1		1		1	1	1		6
Robert J Wilkinson^**[**^ ^**7**^ ^**]**^	1	1	1		1		1	1		6
JiHae Kim^**[**^ ^**6**^ ^**]**^	1	1	1	1	1		1	1		7
J. Jubulis^**[**^ ^**8**^ ^**]**^	1		1	1	1	1	1	1		7
A Sita-Lumsden^**[**^ ^**28**^ ^**]**^	1	1		1	1		1	1		6
Christian Wejse^**[**^ ^**23**^ ^**]**^	1	1	1	1	1	1	1	1		8
PDO Davies^**[**^ ^**26**^ ^**]**^		1	1		1		1	1		5
Lavanya Joshi^**[**^ ^**21**^ ^**]**^		1	1			1	1	1		5
Kara Gray^**[**^ ^**27**^ ^**]**^		1	1		1		1	1		5
HK koo^**[**^ ^**22**^ ^**]**^	1	1	1		1	1	1	1		7
Ginney KB ^**[**^ ^**24**^ ^**]**^			1	1	1		1	1		5
Arnedo-Pena et al 2015^**[**^ ^**29**^ ^**]**^	1		1	1	1		1	1	1	7

Note: “1”represented got 1 star

### Meta-analysis Results

**Fig 2:**
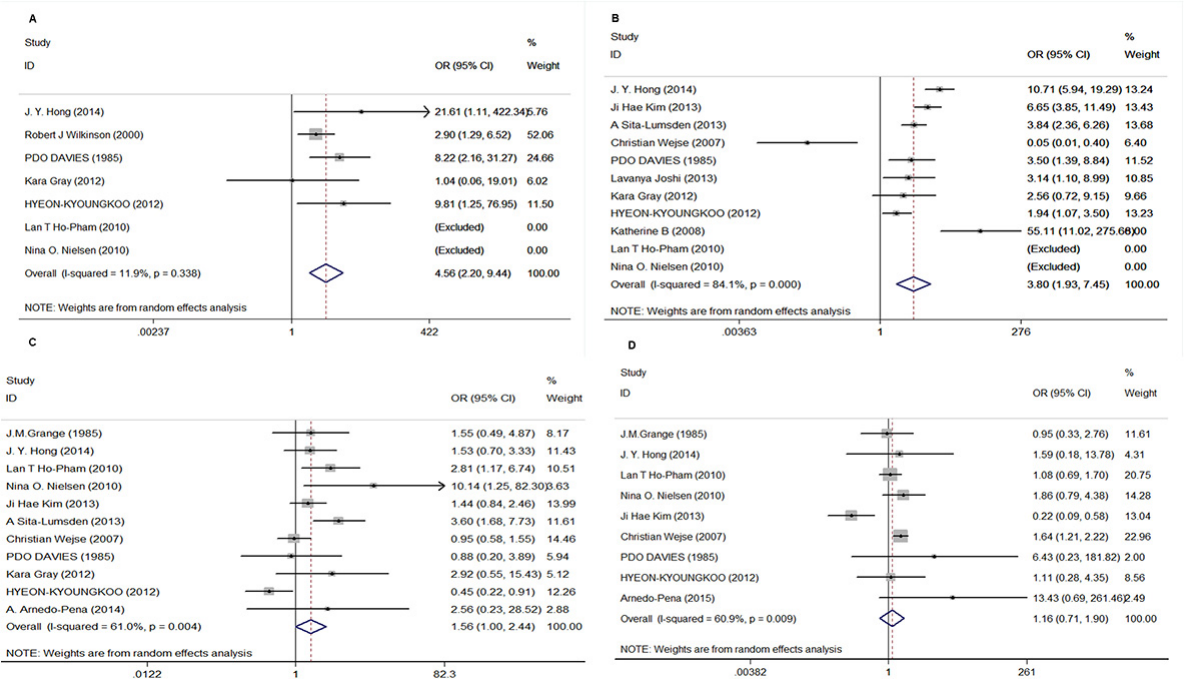
Forest plots of the overall association of susceptibility to tuberculosis and vitamin D deficiency. (a) serum 25(OH)D ≤ 12.5nmol/L; (b) serum 25(OH)D between 12.5 and 25nmol/L; (c) serum 25(OH)D between 26-50nmol/L; (d)serum 25(OH)D between 50 and 75nmol/L.

We obtained the number of subjects in the TB and control groups at different concentration ranges to explore the risk of active TB based on Vitamin D levels. Four concentration ranges were established grounded on original articles: ≤ 12.5 nmol/L, 13–25 nmol/L, 26–50 nmol/L, and 51–75 nmol/L. A significantly increased risk of active TB was identified in serum Vitamin D levels ≤ 12.5 nmol/L (pooled OR = 4.556, 95% CI = 2.200–9.435, I^2^ = 11.9%, *P*<0.001) and 13–25 nmol/L (pooled OR = 3.797, 95% CI = 1.935–7.405, I^2^ = 84.1%,*P*<0.001). Although the pooled OR of levels 26-50nmol/L reached 1.561, the association was not statistically significant. (95%CI = 0.997–2.442, I^2^ = 61.0%,*P* = 0.051). In the 51–75 nmol/L range, no positive association was found (OR = 1.160, 95% CI = 0.708–1.900, I^2^ = 60.9%,*P* = 0.550). These results are presented in Fig 2 and Table 3.

**Table 3:** Results of meta-analyses in different serum Vitamin D ranges.

Serum vitamin D range(nmol/L)	No. of Included study	Number of participants		Pooled ORs	95%CIs	*p*	I^2^
		TB	Control				
≤12.5	7	602	977	4.556	2.200–9.435	<0.001	11.9%
13–25	11	1269	1840	3.797	1.935–7.405	<0.001	84.1%
26–50	10	1244	1794	1.561	0.997–2.442	0.051	61.0%
51–75	8	1052	1428	1.160	0.708–1.900	0.550	60.9%

To determine the effect of an individual study on the summary meta-analysis estimate, we performed sensitivity analyses. A significant change was only founded in the analysis of the range of 26–50 nmol/L. (Table 4) When removing the study by koo HK [22], Davies PD[26] or Wejse C[23], the lower 95% CI varied to be beyond 1.0.

**Table 4:** The results of sensitivity analyses of 26-50nmol/L range.

Study omitted	Estimate	95%CI	
		Lower	Upper
J.M.Grange (1985) *	1. 575	0.969	2.560
J. Y. Hong (2014)*	1.586	0.957	2.630
Lan T Ho-Pham (2010)*	1.455	0.909	2.329
Nina O. Nielsen (2010)*	1.446	0.938	2.231
JiHae Kim (2013)*	1.618	0.949	2.759
A Sita-Lumsden (2013)*	1.365	0.886	2.101
Christian Wejse*	1.714	1.028	2.857
PDO Davies (1985)*	1.629	1.014	2.614
Kara Gray (2012)*	1.512	0.949	2.409
Hyeon-Kyoung koo (2012)	1.775	1.211	2.603
A. Arnedo-Pena (2014)*	1.543	0.971	2.452
Combined	1.561	0.997	2.442

Note:

* studies that significantly influenced the results when omitted.

We next conducted the subgroup analyses by design, population, serum Vitamin D analysis method, and source of controls (random healthy population or tuberculosis contact); however, no significant changes in pooled results or heterogeneity were found. Although pooled OR of level 26-50nmol/L was 1.561, the 95%CI cross 1. Two articles included some participants received anti-TB treatment before vitamin D detection in the concentration range. Considerated the possible influence of anti-TB treatment, we conducted subgroup analysis by treatment status. However, in both untreated group and received treatment group, the results showed no statistically significance in the concentration range.(untreated: OR = 1.570, 95%CI: 0.952–2.588, I^2^ = 59.3%; included treated: OR = 2.491, 0.244–25.442, I^2^ = 79.3%). Meta-regression of publication year and mean age of TB and control group also indicated that none of these factors was a substantial source of heterogeneity. Neither Begg’s test nor Egger’s test found publication bias in any analysis.

These results indicated that a serum Vitamin D concentration ≤ 25 nmol/L was associated with a significantly increased risk of active TB while no positive association was found in level 51–75 nmol/L. Although the pooled OR of level 26–50 nmol/L suggested the positive association, the 95% CI implied no statistically significant in this range. It was also indicated that lower serum vitamin D was associated with higher TB risk (pooled ORs: 4.556 vs. 3.797 vs. 1.561 vs. 1.160). Further investigation is needed because of the high degree of heterogeneity and relatively weak robustness.

## Discussion

A total of 15 studies were included in this analysis. This meta-analysis found that a serum Vitamin D concentration ≤ 25 nmol/L was associated with a significantly increased risk of active TB while the range 51–75 was not associated with active TB. However, the range 26–50 nmol/L was not statistically associated with active TB. Our meta-analysis confirmed the results of a previous meta-analysis that showed that low serum Vitamin D was a risk for active TB and further verified the precise range of low serum Vitamin D posing high risk of TB. To the best of our knowledge, this is the first meta-analysis providing this range.

One recent systematic review reported that mean population-level 25(OH)D3 values varied considerably across the studies (range: 4.9 to 136.2 nmol/L), and more than one-third of the studies reported mean values < 50 nmol/L.[30] In regard to the serum Vitamin D level, a number of different concepts have been proposed, including serum Vitamin D deficiency, serum Vitamin D insufficiency, and normal serum Vitamin D. Some studies assume a value > 75 nmol/L or 80 nmol/L as the normal serum Vitamin D and a value between 50 nmol/L and 75 nmol/L as the serum Vitamin D insufficiency. [9, 25] However, some other studies assume a value < 50 nmol/L as the cutoff for serum Vitamin D insufficiency. [6] Published studies have also used different criteria for the definition of serum Vitamin D deficiency. Some of these studies took the cutoff value of 50 nmol/L,[9, 10] while others used the cutoff value of 25 nmol/L [6–8]. Some other studies used other different and more confusing criteria for the aforementioned concepts. [28,31] A 2008 meta-analysis, which included seven studies, indicated that a low serum Vitamin D level was associated with increased TB risk. [11] In this study, the author obtained the means and standard deviations of serum Vitamin D levels in patients and controls and obtained an “effect size” of the difference in patients and controls. However, the author did not determine the optimal cutoff point or a range of serum Vitamin D levels indicative of an increased risk of TB. Furthermore, since this meta-analysis was published, a large number of studies were conducted during the following years. Thus, we performed this meta-analysis by evaluating the effect of different ranges of serum Vitamin D on active TB to verify the probable high risk range. Based on our results, serum Vitamin D≤ 25nmol/L should be considered to be a susceptibility factor for active TB and 25nmol/L might be a more appropriate cutoff value in the definition of Vitamin D deficiency in TB.

Our results demonstrated lower serum Vitamin D level was associated with higher the risk of active TB. Only serum Vitamin D between 12.5 and 25 nmol/L (95% CI: 1.935–7.405) and ≤ 12.5 nmol/L (95% CI: 2.200–9.435) were associated with a significantly increased risk of active TB. However, 51–75 nmol/L (95% CI: 0.708–1.900) was identified as not associated with an increased risk of active TB. Influence analysis and publication bias analysis confirmed that these results were reliable and robust.

It is interesting that serum vitamin D between 26 nmol/L and 50 nmol/L was not statistically associated with an increased risk of TB according to our analysis. Although the pooled OR was 1.561 in the range 26–50 nmol/L, the 95% CI was cross 1.0 (95% CI: 0.997–2.442, *P* = 0.051). Some original articles included in our study suggested <50nmol/L was the risk level [9,25,28] while some made opposite conclusion[6,10,20,22,23,26,27,29]. Additionally, some prospective studies’ results indicated that vitamin D concentration <50nmol/L increased the risk of TB infection.[17,18] Some explanations might be given to the discrepancy. First of all, factors that impaired the immunity function might influence the determination of the threshold, such as HIV infection. [17] Next, the definition of vitamin D deficiency in Talat et al’ study was 50nmol/L, the different concentration (17.5nmol/L and 32.5nmol/L) was used when compared the level of vitamin D in patients progressed to TB and healthy contact. [18] The exact cutoff value might be less than 50nmol/L in the article. Therefore, we confused the accuracy of 50nmol/L as an appropriate cutoff value. We hypothesized that the optimal value might exist in the range between 25-50nmol/L. However, we could not define it according to available data. In this regard, we concluded that the range of serum Vitamin D levels between 25 nmol/L and 50 nmol/L might pose increased risk of TB but needs further investigation.

Vitamin D deficiency has long been accepted to be associated with impaired immunity and increased risk of TB. [32] Many types of immune cells including monocyte, macrophage, and T-lymphocyte have been proven to play a role in MTB resistance. [4, 33, 34] It was shown that that Vitamin D could induce interleukin-1beta (IL-1β) secretion and further modulated paracrine signaling, which reinforced the role of macrophage in innate immune regulation. [35] Another study noted that Vitamin D could improve the coordinated response to MTB of monocytes and T-lymphocytes in frequent MTB exposure but not in active TB patients. [32] Indeed, most of the studies that investigated the Vitamin D treatment for TB indicated that administration of Vitamin D could not improve clinical outcome among patients with TB. [36, 37] It appears that Vitamin D might primarily play a role in preventing a MTB infection from progressing into active TB and but not curing active TB.

Although Vitamin D has been shown to be immunoregulatory, clinical trials of vitamin D treatment in patients with active tuberculosis have got largely negative results.[38, 39] According to our study, pretreatment serum vitamin D might be another explanation besides different dosing regimens. It might be more efficient to apply vitamin D supplementation for prevention and treatment of tuberculosis when serum vitamin D was lower than 25nmol/L. In this sense, our study provided this possible threshold which might determine the possible benefit individuals and make it more practical. Random controlled trials were needed to verify the results.

There are some limitations in this meta-analysis. The main limitation is the relatively high heterogeneity. Except for the analysis of the < 12.5nmol/L group, the I-squared was > 50% in any other analysis. We attempted to verify the possible source of heterogeneity including study design, population, serum Vitamin D analysis method and source of controls; however, no substantial reduction in heterogeneity was found. In addition, vitamin D receptor genetic polymorphism, vitamin D binding protein, BMI and degree of tuberculosis transmission may be confusion factors. But no enough data could be provided to control them. Although the association between VDR genetic polymorphism and susceptible to TB had been studied for several years, the finding was inconclusive. To determine the role of VDR genetic polymorphism in TB infection, more studies in this field needed to be conducted. Although we could not find the significance of treatment status in our subgroup analysis, anti-TB drugs and other drugs would likely to affect the metabolism of vitamin D. Therefore, we cannot ignore the probable influence, we hope more detail record of the drug use could be obtained to further explore. Second, although 15 studies were included in this meta-analysis, we could not use all of these studies in a single analysis of the four ranges because of incomplete data. This might suggest that future studies could provide an adequate number of participants in different ranges in addition to the mean concentration of serum Vitamin D. Lastly, limited by the nature of retrospective study, we could not clear establish the direction of the association.

Despite the limitations, our meta-analysis has some strong points. First, this is the first meta-analysis to analyze the association between active TB and different ranges of serum Vitamin D. Although previous studies have shown that vitamin D deficiency is a risk factor of tuberculosis, the appropriate range of serum Vitamin D which should be considered risky is not consistent. In Our findings suggest that a serum Vitamin D concentration ≤ 25 nmol/L could be a certain risk factor for active TB, and the lower serum vitamin D may pose higher risk of active TB. Although the heterogeneity was relatively high, the pooled ORs and corresponding 95% CIs were large enough to provide alarming findings. Second, compared to the previous 2008 meta-analysis,[11] this meta-analysis included a far greater number of studies and participants. This facilitates an increased balance of the weight of each study and increases the robustness of the results.

## Conclusions

A serum Vitamin D concentration ≤ 25 nmol/L was significantly associated with an increased risk of active TB but the range 51–75 nmol/L was not associated with an increased risk of TB. The range between 26-50nmol/L might pose the active TB risk. Future well-designed, prospective studies are needed to verify these conclusions.

## Supporting Information

S1 PRISMA Checklist PRISMA 2009 checklist. Preferred Reporting Items for Systematic Reviews and Meta-Analyses
(DOC)

S1 Appendix Newcastle-Ottawa scale. The scoring criteria of the original studies included in our meta-analyses.
(DOC)
